# Machine learning models based on laboratory data: new insight into the differential diagnosis of tuberculous and viral meningitis

**DOI:** 10.3389/fcimb.2026.1791663

**Published:** 2026-04-15

**Authors:** Xiaoyan Hao, Yujiao Hu, Lei Zhou, Fengjuan Wang, Xinran Liu, Jing Wang, Jiankang Chen, Ang Ji, Congxia Bai, Jiayun Liu

**Affiliations:** 1 Department of Clinical Laboratory Medicine, Xijing Hospital, Fourth Military Medical University, Xi’an, China; 2 Department of Neurosurgery, Xijing Hospital, Fourth Military Medical University, Xi’an, China

**Keywords:** diagnosis, machine learning algorithms, nomogram, resampling, tuberculous meningitis, viral meningitis

## Abstract

**Background:**

The differential diagnosis of tuberculous meningitis (TBM) and viral meningitis (VM) remains a formidable clinical challenge. This study aims to develop machine learning (ML) models and a nomogram for differentiating between TBM and VM.

**Methods:**

Clinical and laboratory data were collected from 558 participants, including 190 TBM patients and 368 VM patients, treated between 2000 and 2024 at Xijing Hospital. Resampling techniques were employed to balance the dataset. Four feature selection methods (RFECV-ADA, Boruta, Spearman, MI) were utilized to identify potential indicators. Two supervised ML algorithms were implemented for model development. Model performance was evaluated with the area under the curve, as well as sensitivity (SEN), specificity, accuracy, positive predictive value, and negative predictive value. The influence of each feature was visualized with SHapley Additive exPlanations (SHAP) diagrams. Finally, a nomogram was created from the selected features.

**Results:**

Ten features were identified, including the mean corpuscular volume, hemoglobin, D-Dimer level, cerebrospinal fluid (CSF) glucose, protein, immunoglobulin G, A, M, chloride, and albumin levels. The ENN-XgBoost_V10 model demonstrated the highest SEN of 75.79%, in differentiating TBM from VM and a SEN of 81.45% in patients with at least five leucocytes per μL of CSF and a CSF-to-blood glucose ratio less than 0.5. According to SHAP analysis, the significance of these features in prediction was underscored. Calibration curve analysis indicated that the nomogram predictions were relatively similar to the actual outcomes.

**Conclusions:**

Based on ten routine laboratory tests, the ENN-XgBoost_V10 model differentiates TBM from VM with superior SEN to traditional methods.

## Introduction

Tuberculous meningitis (TBM), a severe cerebral infection caused by Mycobacterium tuberculosis (MTB), is characterized by an initial presentation of nonspecific symptoms, including a vague sense of discomfort, fatigue, headache, and low-grade fever. These early clinical manifestations of TBM are often indistinguishable from those of viral meningitis (VM), resulting in a significant differential diagnostic challenge that impedes prompt etiological diagnosis and treatment and thus negatively affecting patient outcomes. Annually, approximately 50% of individuals affected with TBM either succumb to the disease (including an estimated 78,200 adult fatalities) or suffer from disabilities (Wasserman et al., 2025). Consequently, there is an urgent need for timely therapeutic intervention to avoid mortality from the disease. However, as has been widely acknowledged, diagnosing TBM remains highly challenging.

To date, conventional tests for TBM have been restricted to smear microscopy of cerebrospinal fluid (CSF) and microbiological culture. The former, which include tests such as Ziehl–Neelsen and improved acid–fast staining, tends to have low operational sensitivity (SEN) (Davis and Wilkinson, 2020) (between approximately 3 and 30% (Heemskerk et al., 2018; Park et al., 2016; Feng et al., 2014)), while the latter, despite serving as the gold standard for diagnosing tuberculosis, often takes too long to yield clinically meaningful results. While it is still not widely available in low-income areas, the GeneXpert MTB/RIF Ultra is recommended by the WHO as the initial test for extrapulmonary tuberculosis. When used with CSF, this assay yields a diagnostic SEN for TBM of approximately 40–70% (Huynh et al., 2022; Donovan et al., 2020). Consequently, there is an urgent need for new screening methods that offer increased accessibility and cost-effectiveness.

Conventional laboratory parameters, which are readily accessible in clinical settings, have been extensively used to diagnose, rule out, or monitor a spectrum of diseases. The efficacy and reliability of these parameters in large-scale evaluations are well- established. However, with over 1000 laboratory test items available, manually determining which of them are associated with the progression of TBM is challenging. Machine learning (ML), a pivotal subset of artificial intelligence, offers a solution. ML methods can be grouped into supervised and unsupervised learning, both of which leverage algorithms to enable computers to mine and learn from existing data and allow the construction of predictive models (Camacho et al., 2018). These algorithms utilize feature vectors and corresponding labels to fine-tune the models’ internal parameters, optimization techniques are used to produce the most accurate disease prediction models (Capobianco and Dominietto, 2020). In this way, these algorithms are able to reveal intricate disease patterns that may elude human experts.

Therefore, the aim of the present study was to evaluate the performance of diagnostic models established through ML on the basis of routine laboratory indicators in differentiating TBM from VM.

## Methods

### Study design and data collection

**Figure 1:**
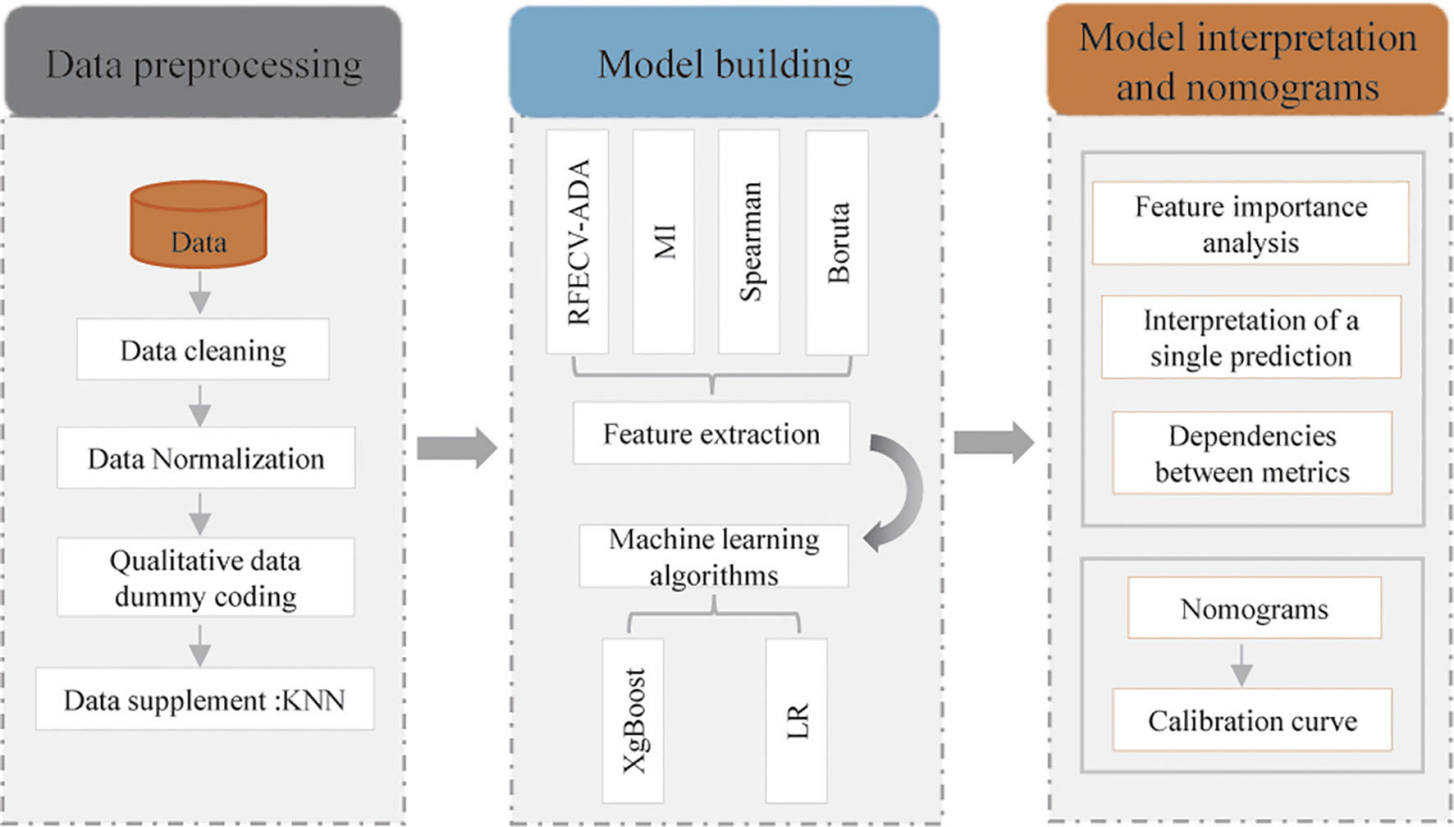
The flow chart of this study.

A retrospective cohort study was conducted at Xijing Hospital in China. The study flowchart is shown in **Figure 1**. Following an initial review of the case records cataloged from January 2000 to July 2024, the following inclusion criteria were applied for TBM patients: 1) positive results from CSF pathogen tests such as acid- fast staining or CSF culture; 2) positive results from CSF molecular biology tests, such as GenXpert, polymerase chain reaction (PCR) or next-generation sequencing (NGS); and 3) a clinical diagnosis of TBM. Similarly, the following inclusion criteria for VM patients were applied: 1) positive molecular results from tests, such as PCR or NGS-based virus detection, for other meningitis-causing viruses, such as human herpesvirus (HHV), enterovirus, and arbovirus, in CSF; and 2) a clinical diagnosis of VM. For both groups, the exclusion criteria were as follows: 1) aged < 18 years and 2) anti-tuberculosis and antiviral treatment for more than 3 days before entering our hospital. The clinical characteristics of the enrolled patients (fever, headache, vomiting, blood pressure, etc.) and laboratory indicator data (routine blood tests, biochemistry, immunity, etc.) were collected from the first examination conducted after the patients arrived at our hospital. The demographic and clinical characteristics of the study population were obtained by consulting the hospital records or medical examination records, and laboratory indicators were obtained by consulting the laboratory information system (LIS).

The study protocol was reviewed and approved by the Human Ethics Committee, Xijing Hospital (Approval No.KY20193292), and the study was conducted in accordance with the principles of Good Clinical Practice and the Declaration of Helsinki. Since it is a retrospective study, the Ethics Committee of Xijing Hospital has waived the requirement for informed consent.

### Data cleaning and normalization

After the raw data were obtained, they were subjected to a number of preprocessing steps. First, indicators for which more than 30% of the data were missing were removed from the dataset. Second, the K-nearest neighbor (KNN) algorithm was used to impute other missing data (Yang et al., 2021) by calculating the distance matrix between data points with missing values and those without missing values, selecting the K data points with the smallest Euclidean distance, and fills the missing values in the data with the field mean corresponding to the selected K-nearest data points.

Next, the fact that each indicator resides in a different dimension could affect the quality of the modeling process. We performed dimensionalization to convert data of different dimensions to the same dimension. We used the MinMaxScaler function of the sklearn.preproduction library to perform interval scaling on the data and normalizes them according to the following formula: vnorm=(v−Vmin)(Vmax−Vmin), where v represents the value of an indicator, $V_{min}$ represents the minimum value of the indicator, and $V_{max}$ represents the maximum value of the indicator.

### Construction of diagnostic models for TBM with different classification algorithms

To identify diagnostic indicators for TBM, four methods were employed to screen differential laboratory indicators, including recursive feature elimination with cross-validation and the AdaBoost classifier (RFECV-ADA), mutual information (MI), Spearman analysis and the Boruta method (Bai et al., 2022; Kong et al., 2022; Bai et al., 2024). The sets of candidate biomarkers identified with the above four methods were then intersected with the scikit learn (v.1.0.1) package and sklearn.model_selection. Next, the KFold function was used to construct two ML classifiers, namely, extreme gradient boosting (XgBoost) and logistic regression (LR) (Hou et al., 2020). Each classifier was used to create a model for distinguishing patients with TBM from those with VM with data from the training set, and the diagnostic performance of these models was validated with the data in the validation set. To ensure model stability and robustness and avoid model overfitting, each model was further evaluated via a tenfold cross-validation resampling technique. Model performance was evaluated using the area under the curve (AUC), SEN, specificity (SPE), accuracy (ACC), positive predictive value (PPV), and negative predictive value (NPV). In addition, a nomogram was constructed through the LR algorithm.

### SHAP-based model explanation

SHapley Additive exPlanations (SHAP) is a Python “Model Interpretation” package (Li et al., 2023) that can interpret the output of any ML model. For each predicted sample, the model generates a predicted value, and the SHAP value is the numerical value assigned to each feature in the sample. Assuming that the i-th sample is $x_{i}$, the j-th feature of the i-th sample is $x_{ij}$, the model’s predicted value for this sample is $y_{i}$, and the baseline of the entire model is $y_{base}$, the SHAP values are determined according to the following equation:


$$y_{i}=y_{base}+f(x_{i1})+\;f(x_{i2})+\ldots +f(x_{ik})$$

where $f(x_{ij})$ is the SHAP value of $x_{ij}$. Intuitively, $f(x_{i1})$ is the contribution of the first feature in the i-th sample to the final predicted value $y_{i}$. When $f(x_{i1})>0$, the feature improves the predicted value and has a positive effect on model performance.

### Statistical analysis

Statistical analysis was performed using SPSS 23.0 (IBM Corp., Armonk, NY, USA). The normality of the sample distribution was determined with the Kolmogorov–Smirnov test. For normally distributed data, differences between groups were compared using the two-tailed unpaired Student’s t-test. For non-normally distributed data, the Mann-Whitney U test with the exact method was used to identify differences between TBM and VM. Categorical variables were compared using the chi-square test or Fisher’s exact test and are presented as percentages. Continuous variables are expressed as mean ± standard deviation or median and interquartile range [M(IQR)]. All tests were two-tailed, and a *P*-value < 0.05 was considered to indicate a statistically significant difference.

## Result

### Characteristics and demographics of the study population

A total of 558 participants were enrolled in the study, including 190 patients with TBM and 368 patients with VM. The clinical characteristics and laboratory indicators whose values were missing in less than 30% of the study cohort are shown in **Table 1**; the missing laboratory indicators are shown in **Supplementary Table 1**. Comparison of the data trends between the imputed dataset and the original dataset demonstrated that the imputation method exhibits good robustness (**Supplementary Table 2**). Clinical features with significant differences between TBM and VM patients included alcohol consumption, meningeal irritation, consciousness, visual disorders, epilepsy, tuberculosis, nutritional status, symptom duration, time from admission to diagnosis, initial CSF pressure, and final CSF pressure. A total of 37 laboratory indicators, including routine blood test, coagulation, routine urine test, and CSF examination indicators and others, were significantly different between TBM patients and VM patients. Additionally, compared with VM patients, TBM patients were more likely to present with meningeal irritation symptoms, longer symptom durations, an elevated CSF pressure, greater diagnostic delay, increased CSF protein levels, and decreased CSF glucose levels. Notably, the mean intervals from hospitalization to diagnosis for VM and TBM patients were 3.82 and 12.01 days, respectively. The predictive model for TBM that this study aimed to create from laboratory indicators could aid in shortening diagnosis timeframes and improving diagnostic SEN.

**Table 1:** Characteristics of patients at baseline and clinical outcomes.

Variables	VM	TBM	*p-*value
	(n=368)	(n=190)	
Age, years ^a^	36.5 (27, 50)	34.5 (24.25, 49)	0.228
Sex ^b^			0.122
Male	247 (67.12%)	115 (60.53%)	
Female	121 (32.88%)	75 (39.47%)	
Dizziness or headache ^b^	333 (90.49%)	173 (91.05%)	0.828
Vomit ^b^	220 (59.78%)	125 (65.79%)	0.166
Epilepsy ^b^	58 (15.76%)	12 (6.32%)	0.001
Impaired consciousness ^b^	68 (18.48%)	64 (33.68%)	<0.001
Language barrier ^b^	71 (19.29%)	48 (25.26%)	0.103
Visual impairment ^b^	41 (11.14%)	47 (24.74%)	<0.001
Blood Pressure ^a^, mmHg			
Diastolic pressure	118 (110, 130)	120 (110, 127.75)	0.635
Systolic pressure	76 (70, 81)	77.5 (70, 80)	0.635
Meningeal irritation ^b^	150 (40.76%)	121 (63.68%)	<0.001
Neck stiffness	115 (31.25%)	108 (56.84%)	<0.001
Kernig	86 (23.37%)	68 (35.80%)	0.002
Brudzinski	47 (12.77%)	42 (22.11%)	0.004
Symptomatic time ^a^, days	14 (8, 30)	20 (10, 30)	<0.001
Interval between admission and diagnosis ^a^, days	1 (0, 3)	4 (2, 9)	<0.001
Smoking ^b^	111 (30.16%)	50 (26.32%)	0.342
Drinking ^b^	86 (23.37%)	30 (15.79%)	0.037
Hypertension ^b^	45 (12.23%)	19 (10%)	0.434
Diabetes ^b^	18 (4.89%)	8 (4.21%)	0.718
Heart disease ^b^	14 (3.80%)	8 (4.21%)	0.815
History of tuberculosis ^b^	6 (1.63%)	20 (10.53%)	<0.001
Nutritional status ^b^			0.022
Malnutrition	14 (3.80%)	17 (8.95%)	
Good Nutrition	352 (95.65%)	173 (91.05%)	
Overnutrition	2 (0.54%)	0 (0.00%)	
Abnormal head MRI ^b^	206 (55.98%)	122 (64.21%)	0.061
Chest radiograph ^b^			0.072
No abnormalities	245 (66.58%)	102 (53.68%)	
Pulmonary nodule	18 (4.89%)	17 (8.95%)	
Inflammation	69 (18.75%)	46 (24.21%)	
Pleural thickening	19 (5.16%)	13 (6.84%)	
Bronchitis	5 (1.36%)	5 (2.63%)	
Cord like structure	9 (2.45%)	4 (2.11%)	
Others	3 (0.82%)	3 (1.58%)	
Blood			
White Blood Cell Count (WBC) ^a^, ×10E9/L	7.01 (5.38, 9.24)	7.34 (5.65, 9.95)	0.11
Neutrophil Ratio^a^, %	0.68 (0.58, 0.78)	0.73 (0.63, 0.84)	<0.001
Lymphocyte Ratio^a^, %	0.23 (0.13, 0.33)	0.16 (0.09, 0.28)	<0.001
Monocyte Ratio^a^, %	0.07 (0.05, 0.09)	0.07 (0.05, 0.09)	0.402
Eosinophil Ratio^a^, %	0.006 (0.001, 0.017)	0.004 (0, 0.013)	0.015
Basophil Ratio^a^, %	0.002 (0.001, 0.004)	0.001 (0, 0.003)	<0.001
Neutrophil Count^a^, ×10E9/L	4.66 (3.15, 7.15)	5.35 (3.74, 8.11)	0.012
Lymphocyte Count^a^, ×10E9/L	1.52 (1.08, 1.91)	1.25 (0.73, 1.8)	<0.001
Monocyte Count^a^, ×10E9/L	0.49 (0.34, 0.67)	0.51 (0.36, 0.76)	0.126
Eosinophil Count^a^, ×10E9/L	0.04 (0.01, 0.1)	0.03 (0, 0.1)	0.026
Basophil Count^a^, ×10E9/L	0.01 (0.01, 0.02)	0.01 (0, 0.02)	0.001
Red Blood Cell Count (RBC) ^a^, ×10E9/L	4.45 (4.07, 4.83)	4.43 (3.99, 4.72)	0.133
Hemoglobin (HGB) ^a^, g/L	137 (127, 149)	131 (119, 145)	0.001
Hematocrit (HCT) ^a^	0.41 (0.38, 0.44)	0.39 (0.36, 0.42)	<0.001
Mean Corpuscular Volume (MCV) ^a^, fL	91.1 (88.3, 94.5)	89.05 (86.1, 92.18)	<0.001
Mean Corpuscular Hemoglobin (MCH) ^a^, pg	30.9 (29.8, 32.1)	30.4 (29, 31.7)	0.002
Mean corpuscular hemoglobin concentration (MCHC) ^a^, g/L	337 (330, 346)	339 (332, 351)	0.04
Red Cell Distribution Width Coefficient of Variation (RDW-CV) ^a^	0.13 (0.12, 0.14)	0.13 (0.13, 0.14)	0.001
Red Cell Distribution Width Standard Deviation (RDW-SD) ^a^, fL	42.6 (40, 45.5)	42 (39.23, 46.3)	0.596
Platelet count (PLT) ^a^, ×10E9/L	204 (169, 258)	225.5 (180, 266.75)	0.1
Platelet Distribution Width (PDW) ^a^, fL	12.4 (11.1, 14.8)	11.65 (10.1, 13.1)	<0.001
Mean Platelet Volume (MPV) ^a^, fL	10.7 (10, 11.7)	10.25 (9.4, 11)	<0.001
Platelet-Larger Cell Ratio (P-LCR) ^a^, %	0.3 (0.25, 0.38)	0.27 (0.2, 0.33)	<0.001
Alanine Aminotransferase (ALT) ^a^, IU/L	24 (16, 40)	24 (16, 39)	0.763
Aspartate Aminotransferase (AST) ^a^, IU/L	20 (15, 27)	20 (14, 29)	0.774
AST/ALT ^a^	0.8 (0.6, 1.2)	0.9 (0.6, 1.2)	0.443
Total Protein ^a^, g/L	64.8 (60.9, 68.55)	64.8 (61.2, 69.4)	0.521
Albumin (ALB) ^a^, g/L	40.4 (37.2, 42.75)	40.4 (37.58, 44.03)	0.373
Globulin (GLO) ^a^, g/L	24.4 (21.98, 27.4)	24.45 (21.48, 27.5)	0.983
Albumin/Globulin (A/G) ^a^	1.6 (1.4, 1.9)	1.7 (1.4, 1.9)	0.922
Total Bilirubin (TBIL) ^a^, μmol/L	12.6 (8.6, 17.8)	12.45 (9.08, 19)	0.451
Direct Bilirubin (DDIL) ^a^, μmol/L	4.7 (3.15, 7)	4.5 (3.5, 7.55)	0.239
Indirect Bilirubin (IDIL) ^a^, μmol/L	7.5 (5.1, 10.8)	7.25 (5.3, 11.63)	0.748
Alkaline Phosphatase (ALP) ^a^, IU/L	67 (55, 84)	66.5 (53, 86.75)	0.992
Gamma-Glutamyl Transferase (GGT) ^a^, IU/L	27 (17, 46)	29 (15, 49.75)	0.89
Urea ^a^, mmol/L	4.4 (3.5, 5.4)	4 (3.2, 5.2)	0.038
Creatinine (Cr) ^a^, μmol/L	82 (73, 93)	76 (67.5, 90.5)	0.001
Uric Acid (UA) ^a^, μmol/L	214 (150.25, 277.75)	150 (104.75, 252.25)	<0.001
Cystatin C (CysC) ^a^, mg/L	0.89 (0.79, 1)	0.86 (0.75, 1)	0.171
Glucose ^a^, mmol/L	5.2 (4.56, 6.34)	5.4 (4.79, 6.44)	0.112
Potassium (K) ^a^, mmol/L	3.99 (3.7, 4.2)	3.9 (3.6, 4.2)	0.393
Sodium (Na) ^a^, mmol/L	139.7 (135.8, 142.03)	136.85 (131.8, 141.28)	0.74
Chloride (Cl) ^a^, mmol/L	102.35 (99.08, 104.93)	101.05 (94.55, 105.15)	0.429
Carbon Dioxide (CO2) ^a^, mmol/L	22.9 (20.7, 25.2)	22.9 (20.6, 25)	0.753
Total Calcium (Ca) ^a^, mmol/L	2.2 (2.1, 2.27)	2.2 (2.1, 2.3)	0.411
Hepatitis B Surface Antigen (HBsAg) ^b^, COI			0.093
–	292 (96.37%)	157 (92.90%)	
+	11 (3.63%)	12 (7.10%)	
Hepatitis B Surface Antibody (anti-HBs) ^b^, mIU/ml			0.48
–	142 (49.65%)	72 (53.33%)	
+	144 (50.35%)	63 (46.67%)	
Hepatitis B e Antigen (HBeAg) ^b^, COI			0.212
–	264 (99.62%)	128 (98.46%)	
+	1 (0.38%)	2 (1.54%)	
Hepatitis B e Antibody (anti-HBe) ^b^, COI			0.353
–	222 (83.77%)	104 (80.00%)	
+	43 (16.23%)	26 (20.00%)	
Hepatitis B Core Antibody (anti-HBc) ^b^, COI			0.267
–	178 (67.68%)	80 (62.02%)	
+	85 (32.32%)	49 (37.98%)	
Human Immunodeficiency Virus Antibody (HIV-Ab) ^b^, S/CO			
–	306 (100.00%)	169 (100.00%)	
+	0 (0.00%)	0 (0.00%)	
High-Sensitivity C-Reactive Protein (hsCRP) ^a^, mg/L	0.17 (0.16, 0.2)	0.17 (0.17, 0.55)	0.104
Prothrombin Time (PT) ^a^, s	11.3 (10.7, 12)	11.6 (10.9, 12.3)	0.013
Activated Partial Thromboplastin Time (APTT) ^a^, s	25.3 (22.3, 28.83)	25 (22.7, 27.95)	0.696
Fibrinogen (FIB) ^a^, g/L	2.95 (2.37, 3.83)	3.08 (2.52, 3.7)	0.312
Thrombin Time (TT) ^a^, s	17.5 (16.3, 18.7)	17.7 (16.1, 18.5)	0.599
D-Dimer ^a^, mg/mL	0.53 (0.25, 2.07)	1.13 (0.47, 8.95)	<0.001
Fibrinogen Degradation Products (FDP) ^a^, μg/ml	1.77 (1.1, 3.96)	2.2 (1.2, 4.45)	0.246
Prothrombin Activity (PTA) ^a^, %	91.3 (83.45, 100.8)	89.9 (79.75, 97.55)	0.022
International Normalized Ratio (INR) ^a^	1 (0.94, 1.04)	1 (0.94, 1.06)	0.335
T.SPOT ^b^			<0.001
–	129 (77.25%)	40 (33.61%)	
+	38 (22.75%)	79 (66.39%)	
Cerebrospinal Fluid (CSF)			
Initial Pressure ^a^, mmH_2_O	165 (130, 220)	235 (170, 300)	<0.001
Final Pressure ^a^, mmH_2_O	100 (75, 130)	120 (90, 150)	<0.001
Protein CSF ^a^, g/L	0.56 (0.37, 0.81)	1.13 (0.6, 1.85)	<0.001
Microalbumin CSF (ALB CSF) ^a^, mg/L	350 (229.5, 511.5)	827 (463.75, 1457.5)	<0.001
Immunoglobulin G (IgG CSF) ^a^, mg/L	48.1 (30.8, 82.5)	140 (67.1, 236.25)	<0.001
Immunoglobulin A (IgA CSF) ^a^, mg/L	6.39 (3.94, 11.9)	23.3 (11.2, 40.325)	<0.001
Immunoglobulin M (IgM CSF) ^a^, mg/L	1.46 (0.61, 3.08)	6.94 (2.66, 13.6)	<0.001
Glucose CSF ^a^, mmol/L	3 (2.64, 3.48)	2.3 (1.6, 2.9)	<0.001
Chloride (Cl CSF) ^a^, mmol/L	121.8 (118.5, 124.5)	115.8 (109.08, 120.2)	<0.001
Acid-Fast Staining ^b^			<0.001
–	368 (100.00%)	142 (74.74%)	
+	0 (0.00%)	48 (25.26%)	
GeneXpert MTB ^b^			<0.001
–	80 (100.00%)	55 (70.51%)	
+	0 (0.00%)	23 (29.49%)	
Urine			
Specific Gravity (SG) ^a^	1.02 (1.02, 1.03)	1.02 (1.02, 1.03)	0.378
pH ^a^	6 (6, 6.5)	6.5 (6, 7)	0.011
Urobilinogen (UBG) ^b^, umol/L			<0.001
–	87 (25.36%)	87 (47.80%)	
±	227 (66.18%)	86 (47.25%)	
+	20 (5.83%)	4 (2.20%)	
++	5 (1.46%)	5 (2.75%)	
+++	4 (1.17%)	0 (0.00%)	
Bilirubin (BIL) ^b^, umol/L			0.574
–	340 (99.13%)	179 (98.35%)	
+	3 (0.87%)	2 (1.10%)	
++	0 (0.00%)	1 (0.55%)	
Nitrites (NIT) ^b^			0.202
–	338 (98.54%)	175 (96.15%)	
+	3 (0.87%)	3 (1.65%)	
++	2 (0.58%)	4 (2.20%)	
Glucose Urine^b^, mmol/L			0.476
–	312 (90.96%)	159 (87.36%)	
±	10 (2.92%)	6 (3.30%)	
+	4 (1.17%)	4 (2.20%)	
++	5 (1.46%)	6 (3.30%)	
+++	7 (2.04%)	6 (3.30%)	
++++	5 (1.46%)	1 (0.55%)	
Ketone (KET) ^b^, mmol/L			0.135
–	267 (77.84%)	149 (81.87%)	
±	16 (4.66%)	9 (4.95%)	
+	38 (11.08%)	14 (7.69%)	
++	12 (3.50%)	7 (3.85%)	
+++	10 (2.92%)	1 (0.55%)	
++++	0 (0.00%)	2 (1.10%)	
Urine Occult Blood^b^,/ul			0.061
–	244 (71.14%)	129 (70.88%)	
±	40 (11.66%)	19 (10.44%)	
+	11 (3.21%)	14 (7.69%)	
++	22 (6.41%)	12 (6.59%)	
+++	22 (6.41%)	4 (2.20%)	
++++	4 (1.17%)	4 (2.20%)	
leukocyte esterase ^b^,/ul			0.87
–	294 (85.71%)	158 (86.81%)	
±	16 (4.66%)	7 (3.85%)	
+	18 (5.25%)	9 (4.95%)	
++	7 (2.04%)	2 (1.10%)	
+++	8 (2.33%)	6 (3.30%)	
Protein Urine ^b^, g/L			0.221
–	256 (74.64%)	148 (81.32%)	
±	52 (15.16%)	21 (11.54%)	
+	28 (8.16%)	8 (4.40%)	
++	6 (1.75%)	5 (2.75%)	
+++	1 (0.29%)	0 (0.00%)	
Urinary White Blood Cell Count (UWBC) ^a^,/ul	7 (2.7, 19.6)	7.6 (3.8, 24.4)	0.145
Urinary Red Blood Cell Count (URBC) ^a^,^/^ul	8 (3.4, 20.75)	9.1 (4.6, 27.9)	0.092
Non-lysed Red Blood Cells ^a^,/ul	5.5 (2.2, 17.9)	6.7 (2.5, 18.25)	0.526
Non-lysed Red Blood Cells Ratio ^a^, %	78.5 (59.35, 92.8)	74.1 (53.1, 88.65)	0.05
Macrocytic Red Blood Cells ^a^,/ul	1.8 (0.5, 6)	1.9 (0.6, 6.4)	0.481
Microcytic Red Blood Cells ^a^,/ul	3.1 (1.35, 8.2)	3.4 (1.7, 9.75)	0.428
Urinary Epithelial Cell Count ^a^,/ul	4.4 (1.9, 11.7)	4.9 (2.1, 15.7)	0.138
Urinary Round Epithelial Cell Count ^a^,/ul	2.5 (0.9, 5.45)	2.9 (1.2, 8.6)	0.078
Urinary Cast Count ^a^,/ul	0.27 (0, 0.93)	0.39 (0, 1)	0.369
Urinary Pathological Cast Count ^a^,/ul	0.13 (0, 0.375)	0.13 (0, 0.36)	0.697
Urinary Bacteria Count ^a^,/ul	17.4 (4.85, 74.8)	28.8 (9.5, 136.8)	0.011
Urinary Crystal Count ^a^,/ul	0.1 (0, 0.4)	0.2 (0, 0.85)	0.043
Urinary Fungal Count ^a^,/ul	0 (0, 0)	0 (0, 0)	0.906
Urinary Mucous Thread Count ^a^,/ul	0.38 (0, 2.285)	0.23 (0, 1.07)	0.007
Others ^a^,/ul	47.7 (23.6, 131.9)	55.2 (29.4, 120)	0.437

The superscript “a” indicates that the data are presented as median and interquartile range [M (P1, P3)]; the superscript “b” indicates that the data are presented as percentages [n (%)].

### Selecting meaningful laboratory indicators as candidate biomarkers for diagnosing TBM

**Figure 2:**
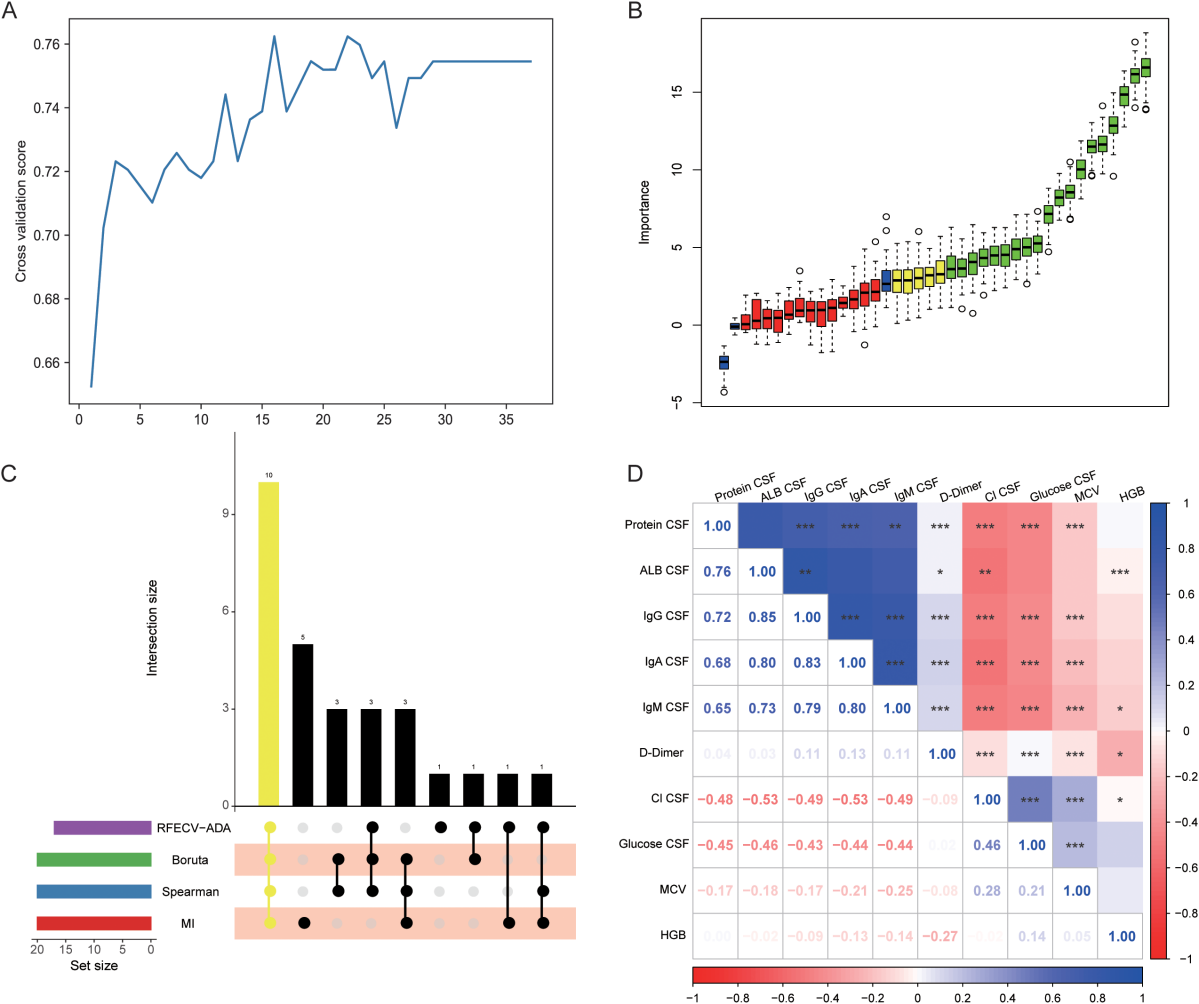
Detection of ten candidate indicators via four classification algorithms. **(A)** RFECV-ADA was used to screen candidate indicators. **(B)** The Boruta algorithm was used to candidate indicators. **(C)** Venn diagram showing the intersection of the indicators obtained with the four algorithms (RFECV-ADA, Boruta, MI, Spearman). **(D)** Heatmaps between candidate indicators. RFECV-ADA, Adaptive Boosting recursive feature elimination; MI, mutual information. *,** , and *** represent p-values less than 0.05, 0.01, and 0.001, respectively.

To further screen the 37 differential laboratory indicators to identify diagnostic biomarkers for TBM, we used the RFECV-ADA, MI, Spearman, and Boruta algorithms to rank the importance of the indicators on the basis of their values across all samples. Considering the class imbalance in the dataset, which would affect the performance of the algorithm, we adopted undersampling processing (edited nearest neighbors, ENN) on the dataset to ensure a class ratio of 1:1 (190 patients per disease). We identified 16 indicators with the highest scores from RFECV-ADA (**Figure 2A**), whereas the Boruta algorithm identified 19 important indicators (**Figure 2B**). By intersecting these features with the top 20 indicators from the MI and Spearman methods (**Supplementary Table 3**), we ultimately identified 10 indicators as candidate biomarkers for diagnosing TBM and further evaluated their diagnostic value (**Figure 2C**). These ten indicators include the mean corpuscular volume (MCV), hemoglobin (HGB), level of D-Dimer in the blood, and the levels of glucose, protein, immunoglobulin G (IgG), immunoglobulin A (IgA), immunoglobulin M (IgM), chloride (Cl), and albumin (ALB) in the CSF. Spearman correlation analysis was subsequently conducted to assess the correlations among these candidate biomarkers. The CSF IgG, IgA, and IgM were strongly positively correlated with each other (p < 0.001) but negatively correlated with CSF Cl and glucose and MCV (p < 0.001) (**Figure 2D**).

### The ENN-XgBoost_V10 model had the best performance in diagnosing TBM

**Figure 3:**
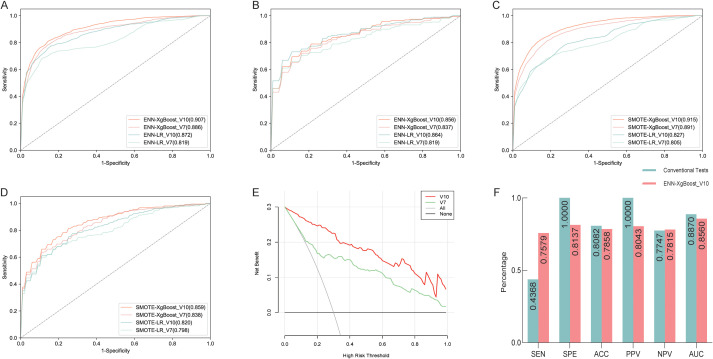
Diagnostic value of the candidate indicators in tuberculous meningitis patients. **(A)** Generate Receiver Operating Characteristic (ROC) curves for the training sets of two models under undersampling using the values of 10 candidate indicators to distinguish between TBM from viral meningitis (VM). **(B)** Generate Receiver Operating Characteristic (ROC) curves for the validation sets of two models under undersampling using the values of 10 candidate indicators to distinguish between TBM from VM. **(C)** Generate Receiver Operating Characteristic (ROC) curves for the training sets of two models under oversampling using the values of 10 candidate indicators to distinguish between TBM from VM. **(D)** Generate Receiver Operating Characteristic (ROC) curves for the validation sets of two models under oversampling using the values of 10 candidate indicators to distinguish between TBM from VM. **(E)** DCA curves of 10 candidate indicators and 7 CSF candidate indicators were generated to differentiate TBM from VM. **(F)** Performance Comparison between ENN-XgBoost_V10 Model and conventional detection.

To further assess the diagnostic efficacy of the candidate biomarkers for TBM, we constructed two ML classification models, named ENN-XgBoost_V10 and ENN-LR_V10, on the basis of the above 10 candidate biomarkers in the training cohort (342 patients, 90%). Model performance was subsequently validated in a validation cohort (38 patients, 10%) as well as tenfold cross-validation in the entire cohort; the results are shown in **Table 2** and **Figure 3**. The results of each evaluation metric are presented as the mean values derived from 10-fold cross-validation.

**Table 2:** Performance of model.

Model	Sets	SEN	SPE	ACC	PPV	NPV	AUC
ENN-XgBoost_V10	Training set	78.48%	87.10%	82.83%	85.70%	80.45%	0.907 (0.876-0.938)
	Validation set	75.79%	81.37%	78.58%	80.43%	78.15%	0.856 (0.818-0.894)
ENN-LR_V10	Training set	71.11%	88.89%	80.07%	86.30%	75.76%	0.872 (0.836-0.908)
	Validation set	69.47%	87.55%	78.58%	85.68%	75.16%	0.864 (0.827-0.901)
SMOTE-XgBoost_V10	Training set	81.88%	85.63%	83.76%	85.07%	82.54%	0.915 (0.894-0.936)
	Validation set	76.07%	79.09%	77.58%	78.52%	77.58%	0.859 (0.832-0.886)
SMOTE-LR_V10	Training set	65.82%	83.09%	74.46%	79.56%	70.86%	0.827 (0.797-0.857)
	Validation set	65.76%	83.15%	74.46%	79.25%	71.24%	0.820 (0.789-0.851)
ENN-XgBoost_V7	Training set	77.49%	86.07%	81.81%	84.61%	79.55%	0.886 (0.852-0.92)
	Validation set	72.63%	81.84%	77.27%	81.30%	76.52%	0.837 (0.796-0.878)
ENN-LR_V7	Training set	66.73%	89.52%	78.21%	86.24%	73.22%	0.819 (0.776-0.862)
	Validation set	65.26%	89.68%	77.55%	86.81%	73.19%	0.819 (0.776-0.862)
SMOTE-XgBoost_V7	Training set	77.75%	84.66%	81.20%	83.53%	79.19%	0.891 (0.867-0.915)
	Validation set	70.38%	80.19%	75.28%	77.75%	73.60%	0.838 (0.809-0.867)
SMOTE-LR_V7	Training set	64.67%	85.14%	74.91%	81.32%	70.68%	0.805 (0.773-0.837)
	Validation set	64.68%	84.50%	74.59%	80.43%	70.89%	0.798 (0.766-0.83)

SEN, sensitivity; SPE, specificity; ACC, accuracy; PPV, positive predictive value; NPV, negative predictive value; AUC, area under curves.

We found that the ENN-XgBoost_V10 model had the highest SEN (75.79% in the validation set), whereas the ENN-LR_V10 classifier had the highest SPE (87.55% in the validation set). The AUCs of both models in both the training and validation sets were greater than 0.85 (**Figures 3A, B**). Next, to further verify the reusability of the model, we employed the synthetic minority oversampling technique (SMOTE) on the original dataset, producing new TBM and VM groups with data from 368 “patients” each. The resulting SMOTE-based models also demonstrated good performance, especially the SMOTE-XgBoost_V10 model, which yielded more stable results than the SMOTE-LR_V10 model, with a SEN, SPE, and AUC in the validation set of 76.07%, 79.09%, and 0.859, respectively (**Table 2**; **Figures 3C, D**). Additionally, we have supplemented the standard deviations of the 10-fold cross-validation results (**Supplementary Table 4**). In addition, we also established XgBoost models using 178 viral meningitis cases and 178 tuberculous meningitis cases that were completely excluded from feature extraction. The performance of these models is summarized in **Supplementary Table 5**. The results demonstrated that the model performance was consistent with previously observed trends, further confirming the robustness of our models.

Our analysis revealed that among the 10 candidate biomarkers, three were blood indicators, and seven were CSF indicators. Regardless of the resampling method used, the performance of the model based on only seven CSF indicators was generally lower than that of the model based on all ten indicators (**Table 2**; **Figures 3A–D**). To further assess the clinical utility of these 10 indicators and compare it with that of the subset of 7 CSF indicators, we conducted decision curve analysis (DCA) of the models based on the two sets of indicators. Consistent with our expectations, the DCA results demonstrated that the net benefits of the model incorporating all 10 indicators exceeded those of the model based solely on the 7 CSF indicators across all thresholds (**Figure 3E**). These findings suggest that the model constructed with the comprehensive set of 10 candidate biomarkers has superior clinical utility than the CSF indicator-based model.

### The SEN of the model surpasses that of the gold standard for diagnosing TBM

At present, conventional methods for diagnosing TBM include pathogen and molecular diagnostic techniques. We conducted a comparative analysis of the performance of the ENN-XgBoost_V10 model with that of these conventional diagnostic methods for TBM (**Figure 3F**). Compared with traditional diagnostic methods, the ENN-XgBoost_V10 model exhibited markedly greater SEN (75.79% versus 43.68%) and a marginally improved NPV (78.15% versus 77.47%). Conversely, the SPE, ACC, PPV, and AUC of the ENN-XgBoost_V10 model were slightly lower than those of the conventional methods.

### Importance and dependence of candidate biomarkers

**Figure 4:**
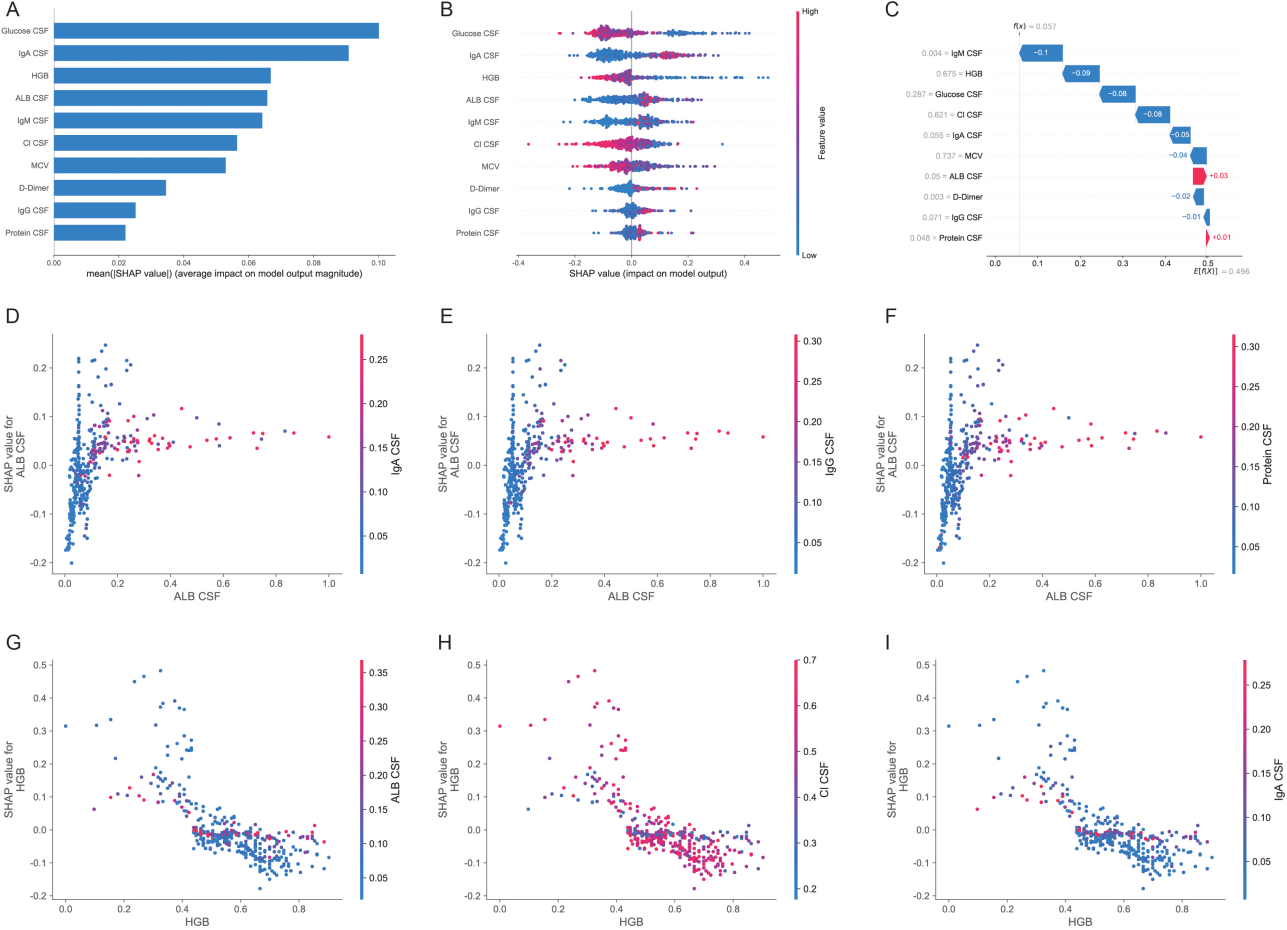
SHAP diagram was used for model interpretation. **(A)** Bar chart for ten candidate indicators. **(B)**Bee swarm plots for ten candidate indicators. **(C)** Explanation of a single indicator. **(D–I)** Dependency graph between indicators **(D)** ALB CSF vs IgA CSF **(E)** ALB CSF vs IgG CSF **(F)** ALB CSF vs Protein CSF **(G)** HGB vs ALB CSF **(H)** HGB vs Cl CSF **(I)** HGB vs IgA CSF.

To visually explain the importance of the selected biomarkers, we used SHAP visualization to illustrate how these biomarkers affect the performance of the model (**Figure 4**). The higher the overall SHAP value is, the more the variable contributes to the prediction of the model. We evaluated the specific contributions of the candidate indicators to a particular prediction (**Figure 4A**). Moreover, we visualized the SHAP values for each feature of every sample (**Figure 4B**). Here, individuals with higher glucose CSF values (represented in red) are more likely to be diagnosed with VM than are individuals with lower values (represented in blue) (left); additionally, individuals with lower CSF IgA values (represented in blue) are more likely to be diagnosed with VM than are individuals with higher test values (represented in red) (right). SHAP values show the contribution of each feature to the final prediction and can effectively clarify and explain model predictions for individual patients. We have provided another example to illustrate the interpretability of the model (**Figure 4C**). Furthermore, we also plotted the dependencies between the indicators (**Figures 4D–I**). For example, CSF ALB has a smaller effect on the predictions for individuals with higher CSF IgA values (**Figure 4D**), whereas HGB has a greater effect on the predictions for individuals with higher CSF Cl values (**Figure 4H**).

### The model demonstrates superior performance in identifying patients with suspected TBM

Clinically suspected TBM was defined as the presence of at least five leucocytes per microliter of CSF and a CSF-to-blood glucose ratio less than 0.5 (van Laarhoven et al., 2018). According to these criteria, 104 TBM patients and 96 VM were considered to have clinically suspected TBM. The SEN, SPE, and ACC of the XgBoost model in this new cohort were 81.45%, 75.44%, and 78.50%, respectively, whereas those of the gold standard were 50.96%, 100%, and 74.5%, respectively. These findings indicate that the ML model is a promising noninvasive and rapid diagnostic tool for the identification of patients with suspected TBM.

### Development and validation of a prediction nomogram

**Figure 5:**
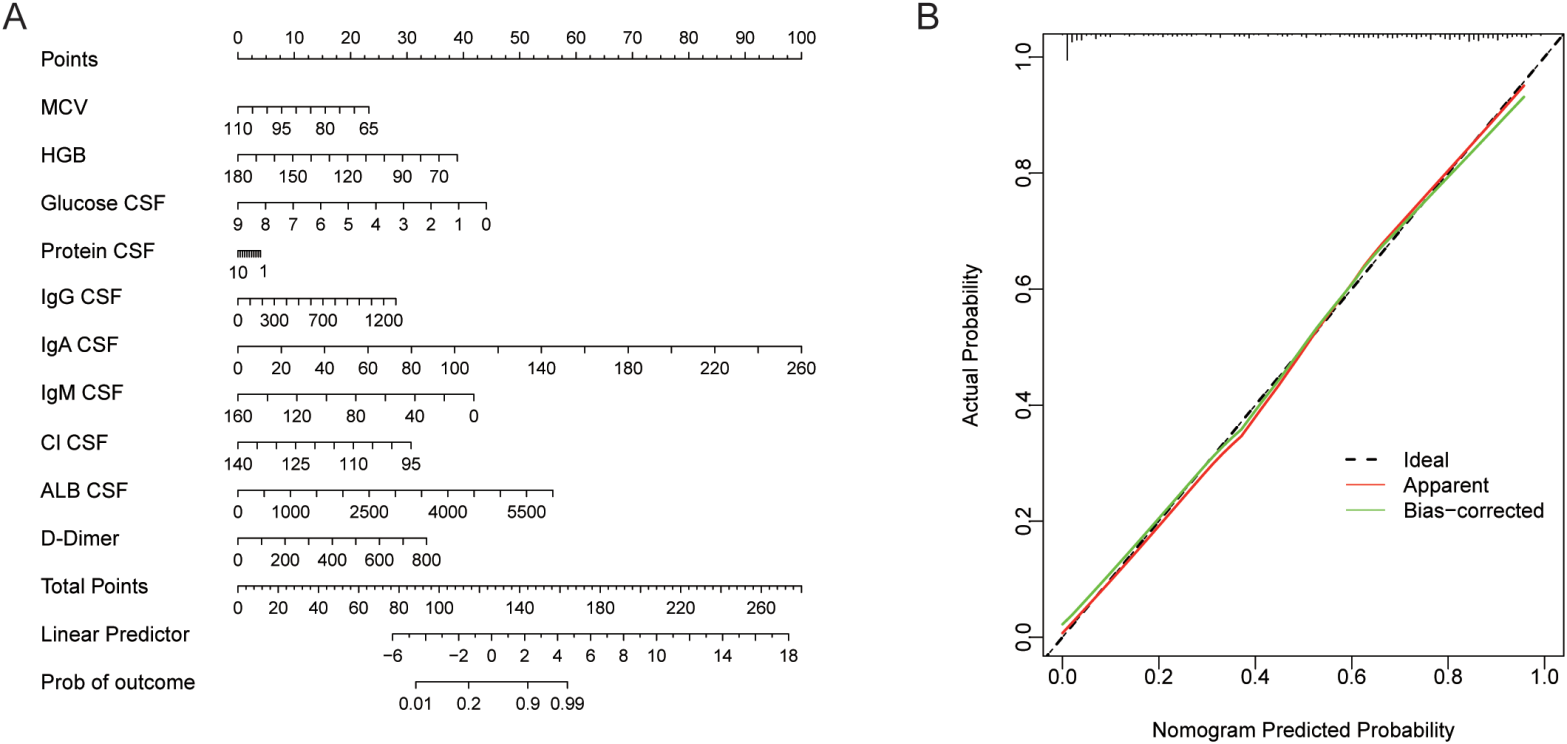
Construction and verification of nomogram. **(A)** Nomogram to predict TBM. **(B)** Calibration curves of the predicted nomogram.

Nomograms are widely accepted as reliable tools for predicting outcomes at the individual level. Each variable in the prediction model is assigned a score, and the sum of the scores for all variables for an individual is matched to a predicted probability (**Figure 5A**). The calibration curve of the nomogram closely aligns with the ideal diagonal line (**Figure 5B**), indicating a high degree of ACC in the model’s predictive performance.

## Discussion

In this study, we developed two classification models, ENN-XgBoost_V10 and ENN-LR_V10, on the basis of routine laboratory test results. Despite their basis on only ten indicators, these models, particularly the ENN-XgBoost_V10 model, exhibited excellent SEN in distinguishing TBM patients from VM patients.

Specifically, after ENN undersampling processing, 10 indicators, including 2 routine blood test indicators, 1 coagulation indicator, and 7 CSF indicators, were jointly identified through the use of the RFECV-ADA, MI, Spearman, and Boruta methods. The causes of anemia are clinically diverse, mainly including chronic consumptive diseases such as tuberculosis, human immunodeficiency virus (HIV) infection, malaria, hematologic malignancies, and various nutritional deficiencies (Strand et al., 2014). Early identification of such patients through the measurement of HGB levels may result in closer monitoring and a reduction in mortality. As a measure of the mean size of erythrocytes, the MCV is a useful index for diagnosing infectious diseases (Liang et al., 2023). Previous studies have reported that low MCV values are associated with increased risks of mortality and infection (Honda et al., 2020). Another study reported an association between coagulation dysfunction and MTB infection (Turken et al., 2002). A retrospective case study revealed significantly increased serum D-Dimer levels in patients with tuberculosis-associated ischemic stroke. Systemic coagulation pathways are activated in patients with pulmonary TB, whereas systemic anticoagulant pathways are downregulated (Wei et al., 2022). Antibodies such as IgG, IgA and IgM produced against MTB antigens have been reported in the CSF of patients with TBM, and thus *in vitro* diagnostics of these items have been used for the early diagnosis of TBM (Sardella et al., 2010; Park et al., 1993; He et al., 2024). In our study, similar to previous studies, patients with TBM had significantly higher levels of CSF IgG, IgA and IgM than those with VM did. Mycobacterium tuberculosis consumes glucose in the CSF while producing lactic acid and pyruvate, all of which result in a reduced glucose content in the CSF (Zhong et al., 2017; van Zyl et al., 2020). In TBM, a low level of glucose is correlated with more severe disease, possibly due to higher mycobacterial loads, resulting in increased bacterial detection (Heemskerk et al., 2018). The elevated CSF protein level in TBM can be attributed to inflammatory cell exudation, as the tuberculous exudate contains monocytes, lymphocytes, and cellulose, all of which contribute to increased protein levels (Luo et al., 2020). Metabolic acid production by MTB reduces the pH of the CSF, which in turn affects the chloride content (Mason and Solomons, 2021). When there is an increase in protein in the CSF, the chloride content is relatively reduced to maintain the Donnan balance, often in conjunction with the glucose reduction (Kurbel, 2011). Thus, a decrease in the CSF chloride concentration is an important feature of a TBM.

We employed supervised ML algorithms to evaluate the selected candidate indicators as predictive factors for the TBM. We observed that the ENN-XgBoost_V10 model had the best performance in diagnosing TBM, with a SEN of 75.79%. After applying SMOTE to artificially balance the samples, the SEN was 76.07%. However, the current gold standard SEN is only 43.68%, indicating that the constructed model maintained a clear advantage. In addition, we verified that the model constructed with only the 7 CSF indicators performed worse than that constructed with all 10 indicators. Moreover, in subgroup analysis, the SEN of the XgBoost_V10 model reached 81.45%. Taken together, these findings demonstrate the reliability of the constructed model from multiple perspectives.

In terms of feature importance, we expected the CSF-related indicators to be ranked highly. Surprisingly, however, HGB ranked third in feature importance, with lower HGB values being more indicative of a diagnosis of TBM. HGB is a hematological index that has been reported to be associated with bacterial infection (Tan et al., 2021). Tuberculous meningitis has an insidious onset and a prolonged course, with typical neurological symptoms gradually emerging over days to weeks (Donovan et al., 2026). This chronic consumptive process provides a sufficient time window for the development of anemia resulting from persistently enhanced systemic catabolism and malnutrition. In contrast, 88% of viral meningitis cases present with acute onset and a short, self-limiting course (Tyler, 2018), and thus lack the pathophysiological basis for the development of anemia described above. However, the absence of a fungal meningitis control group represents a limitation of the present study. In future work, we will include fungal meningitis for comparative studies.

In this study, the ENN-XgBoost_V10 model was primarily developed as a screening tool to facilitate the early identification of TBM among patients with suspected meningitis. Given the challenges associated with rapid and sensitive microbiological diagnosis of TBM, a model with reasonably high sensitivity can help flag high-risk individuals for prompt empirical anti-tuberculosis therapy or further confirmatory testing. We acknowledge that the specificity of 81% yields a false-positive rate of nearly 20%, which may lead to unnecessary treatment or investigation in some patients with VM. However, in the context of TBM, where delays in treatment can be life-threatening, the trade-off between sensitivity and specificity may be acceptable if the model is used as an initial screening step rather than a standalone diagnostic tool. We have also suggested that, in clinical practice, patients identified as high-risk by the model should undergo further confirmatory testing (e.g., microbiological or molecular assays) before treatment initiation.

However, several limitations to this study should be acknowledged. First, this model lacks external validation as it was developed solely based on data from a single center. Although various resampling strategies were employed to evaluate the model, these internal validations cannot fully substitute for external validation in independent populations or temporal cohorts. Therefore, caution should be exercised when generalizing our findings to other healthcare settings or patient populations from different time periods. In future work, we aim to collect multi-center big data to externally validate this model. Second, in the future, data from patients with bacterial meningitis should be included to further verify the efficacy of the screening indicators in differentiating bacterial meningitis from TBM. Third, this study has several limitations regarding missing data. Although we assumed a Missing At Random (MAR) mechanism and applied KNN imputation, the data were not Missing Completely At Random (MCAR). In future work, we will pay greater attention to the missingness mechanisms of missing data and the imputation strategies employed.

## Conclusion

In conclusion, the main purpose of our paper was to explore diagnostic indicators for TBM. Feature selection methods were used to create an optimal feature subset. The application of ML algorithms could help guide the development of more personalized and effective methods for diagnosing TBM.

## Data Availability

The original contributions presented in the study are included in the article/**Supplementary Material**. Further inquiries can be directed to the corresponding authors.
