# Supplementary material for: Machine learning models based on laboratory data: new insight into the differential diagnosis of tuberculous and viral meningitis
Source: Front Cell Infect Microbiol. 2026 Apr 15;16:1791663. doi: 10.3389/fcimb.2026.1791663 (PMC13125095; doi:10.3389/fcimb.2026.1791663)
Supplement: Supplementary file 1 [file Table1.docx]

**Supplementary Table 1: Proportion of Missing Laboratory Indicators**

| Variatables | Total Missing Subjects | Missing Cases of Viral Meningitis |
| --- | --- | --- |
| Sex | 0(0%) | 0(0%) |
| Age | 0(0%) | 0(0%) |
| White Blood Cell Count (WBC) | 7(1.25%) | 7(1.9%) |
| Neutrophil Ratio | 7(1.25%) | 7(1.9%) |
| Lymphocyte Ratio | 7(1.25%) | 7(1.9%) |
| Monocyte Ratio | 7(1.25%) | 7(1.9%) |
| Eosinophil Ratio | 7(1.25%) | 7(1.9%) |
| Basophil Ratio | 7(1.25%) | 7(1.9%) |
| Neutrophil Count | 7(1.25%) | 7(1.9%) |
| Lymphocyte Count | 7(1.25%) | 7(1.9%) |
| Monocyte Count | 7(1.25%) | 7(1.9%) |
| Eosinophil Count | 7(1.25%) | 7(1.9%) |
| Basophil Count | 7(1.25%) | 7(1.9%) |
| Red Blood Cell Count (RBC) | 7(1.25%) | 7(1.9%) |
| Hemoglobin (HGB) | 7(1.25%) | 7(1.9%) |
| Hematocrit (HCT) | 7(1.25%) | 7(1.9%) |
| Mean Corpuscular Volume (MCV) | 7(1.25%) | 7(1.9%) |
| Mean Corpuscular Hemoglobin (MCH) | 7(1.25%) | 7(1.9%) |
| Mean corpuscular hemoglobin concentration (MCHC) | 7(1.25%) | 7(1.9%) |
| Red Cell Distribution Width Coefficient of Variation (RDW-CV) | 7(1.25%) | 7(1.9%) |
| Red Cell Distribution Width Standard Deviation (RDW-SD) | 7(1.25%) | 7(1.9%) |
| Platelet count (PLT) | 7(1.25%) | 7(1.9%) |
| Platelet Distribution Width (PDW) | 14(2.51%) | 12(3.26%) |
| Mean Platelet Volume (MPV) | 14(2.51%) | 12(3.26%) |
| Platelet-Larger Cell Ratio (P-LCR) | 14(2.51%) | 12(3.26%) |
| Alanine Aminotransferase (ALT) | 19(3.41%) | 13(3.53%) |
| Aspartate Aminotransferase (AST) | 17(3.05%) | 12(3.26%) |
| AST/ALT | 19(3.41%) | 13(3.53%) |
| Total Protein | 19(3.41%) | 13(3.53%) |
| Albumin (ALB) | 19(3.41%) | 13(3.53%) |
| Globulin (GLO) | 22(3.94%) | 16(4.35%) |
| Albumin/Globulin (A/G) | 19(3.41%) | 13(3.53%) |
| Total Bilirubin (TBIL) | 19(3.41%) | 13(3.53%) |
| Direct Bilirubin (DDIL) | 29(5.2%) | 17(4.62%) |
| Indirect Bilirubin (IDIL) | 29(5.2%) | 17(4.62%) |
| Alkaline Phosphatase (ALP) | 29(5.2%) | 17(4.62%) |
| Gamma-Glutamyl Transferase (GGT) | 29(5.2%) | 17(4.62%) |
| Urea | 18(3.23%) | 15(4.08%) |
| Creatinine (Cr) | 18(3.23%) | 15(4.08%) |
| Uric Acid (UA) | 48(8.6%) | 34(9.24%) |
| Cystatin C (CysC) | 23(4.12%) | 17(4.62%) |
| Glucose | 22(3.94%) | 10(2.72%) |
| Potassium (K) | 14(2.51%) | 12(3.26%) |
| Sodium (Na) | 14(2.51%) | 12(3.26%) |
| Chloride (Cl) | 14(2.51%) | 12(3.26%) |
| Carbon Dioxide (CO2) | 14(2.51%) | 12(3.26%) |
| Total Calcium (Ca) | 14(2.51%) | 12(3.26%) |
| Hepatitis B Surface Antigen (HBsAg) | 86(15.41%) | 65(17.66%) |
| Hepatitis B Surface Antibody (anti-HBs) | 137(24.55%) | 82(22.28%) |
| Hepatitis B e Antigen (HBeAg) | 163(29.21%) | 103(27.99%) |
| Hepatitis B e Antibody (anti-HBe) | 163(29.21%) | 103(27.99%) |
| Hepatitis B Core Antibody (anti-HBc) | 166(29.75%) | 105(28.53%) |
| Human Immunodeficiency Virus Antibody (HIV-Ab) | 83(14.87%) | 62(16.85%) |
| High-Sensitivity C-Reactive Protein (hsCRP) | 155(27.78%) | 78(21.2%) |
| Prothrombin Time (PT) | 71(12.72%) | 52(14.13%) |
| Activated Partial Thromboplastin Time (APTT) | 71(12.72%) | 52(14.13%) |
| Fibrinogen (FIB) | 71(12.72%) | 52(14.13%) |
| Thrombin Time (TT) | 73(13.08%) | 53(14.4%) |
| D-Dimer | 82(14.7%) | 54(14.67%) |
| Fibrinogen Degradation Products (FDP) | 82(14.7%) | 55(14.95%) |
| Prothrombin Activity (PTA) | 71(12.72%) | 52(14.13%) |
| International Normalized Ratio (INR) | 71(12.72%) | 52(14.13%) |
| Protein CSF | 25(4.48%) | 12(3.26%) |
| Microalbumin CSF | 66(11.83%) | 30(8.15%) |
| Immunoglobulin G (IgG CSF ) | 45(8.06%) | 20(5.43%) |
| Immunoglobulin A (IgA CSF ) | 44(7.89%) | 19(5.16%) |
| Immunoglobulin M (IgM CSF) | 45(8.06%) | 20(5.43%) |
| Glucose CSF | 54(9.68%) | 39(10.6%) |
| Chloride(Cl CSF) | 63(11.29%) | 29(7.88%) |
| Specific Gravity (SG) | 34(6.09%) | 26(7.07%) |
| pH | 33(5.91%) | 25(6.79%) |
| Urobilinogen (UBG) | 33(5.91%) | 25(6.79%) |
| Bilirubin (BIL) | 33(5.91%) | 25(6.79%) |
| Nitrites (NIT) | 33(5.91%) | 25(6.79%) |
| Glucose Urine | 33(5.91%) | 25(6.79%) |
| Ketone (KET) | 33(5.91%) | 25(6.79%) |
| Urine Occult Blood | 33(5.91%) | 25(6.79%) |
| leukocyte esterase | 33(5.91%) | 25(6.79%) |
| Protein Urine | 33(5.91%) | 25(6.79%) |
| Urinary White Blood Cell Count (UWBC) | 34(6.09%) | 25(6.79%) |
| Urinary Red Blood Cell Count (URBC) | 34(6.09%) | 25(6.79%) |
| Non-lysed Red Blood Cells | 84(15.05%) | 69(18.75%) |
| Non-lysed Red Blood Cells Ratio | 84(15.05%) | 69(18.75%) |
| Macrocytic Red Blood Cells | 84(15.05%) | 69(18.75%) |
| Microcytic Red Blood Cells | 84(15.05%) | 69(18.75%) |
| Urinary Epithelial Cell Count | 34(6.09%) | 25(6.79%) |
| Urinary Round Epithelial Cell Count | 52(9.32%) | 41(11.14%) |
| Urinary Cast Count | 34(6.09%) | 25(6.79%) |
| Urinary Pathological Cast Count | 52(9.32%) | 41(11.14%) |
| Urinary Bacteria Count | 34(6.09%) | 25(6.79%) |
| Urinary Crystal Count | 52(9.32%) | 41(11.14%) |
| Urinary Fungal Count | 52(9.32%) | 41(11.14%) |
| Urinary Mucous Thread Count | 52(9.32%) | 41(11.14%) |
| Others | 53(9.5%) | 41(11.14%) |

**Supplementary Table 2: Comparison between imputed data and** **original data**

|  | before impute | | | after impute | | |
| --- | --- | --- | --- | --- | --- | --- |
|  | Median | Q25% | Q75% | Median | Q25% | Q75% |
| Mean Corpuscular Volume (MCV) | 90.3 | 87.45 | 93.7 | 90.35 | 87.6 | 93.7 |
| Lymphocyte Count | 1.45 | 1 | 1.885 | 1.45 | 1 | 1.88 |
| Mean corpuscular hemoglobin concentration | 338 | 330.5 | 348 | 338 | 330.1 | 348 |
| Hematocrit (HCT) | 0.401 | 0.365 | 0.432 | 0.4 | 0.36515 | 0.432 |
| Lymphocyte Ratio | 0.206 | 0.1175 | 0.3085 | 0.2105 | 0.11825 | 0.30775 |
| Hemoglobin (HGB) | 135 | 124 | 148 | 135 | 124 | 148 |
| Neutrophil Ratio | 0.7 | 0.599 | 0.8065 | 0.6995 | 0.6 | 0.80425 |
| Mean Corpuscular Hemoglobin (MCH) | 30.8 | 29.5 | 31.95 | 30.8 | 29.6 | 31.9 |
| Neutrophil Count | 4.88 | 3.26 | 7.345 | 4.895 | 3.275 | 7.325 |
| Red Cell Distribution Width Coefficient of Variation (RDW-CV) | 0.13 | 0.123 | 0.139 | 0.13 | 0.123 | 0.139 |
| Basophil Count | 0.01 | 0.01 | 0.02 | 0.01 | 0.01 | 0.02 |
| Basophil Ratio | 0.002 | 0.001 | 0.003 | 0.002 | 0.001 | 0.003 |
| Eosinophil Count | 0.04 | 0.01 | 0.1 | 0.04 | 0.01 | 0.1 |
| Eosinophil Ratio | 0.005 | 0.001 | 0.016 | 0.0055 | 0.001 | 0.016 |
| Platelet-Larger Cell Ratio (P-LCR) | 0.29 | 0.229 | 0.36325 | 0.292 | 0.2305 | 0.361 |
| Mean Platelet Volume (MPV) | 10.6 | 9.8 | 11.5 | 10.6 | 9.9 | 11.4 |
| Platelet Distribution Width (PDW) | 12.2 | 10.8 | 14.2 | 12.3 | 10.8 | 14.2 |
| Creatinine (Cr) | 81 | 70 | 92 | 81 | 71 | 92 |
| Urea | 4.3 | 3.4 | 5.3 | 4.3 | 3.4 | 5.315 |
| Glucose CSF | 2.9 | 2.4 | 3.38 | 2.92 | 2.4 | 3.3 |
| Protein CSF | 0.69 | 0.4 | 1.1 | 0.6 | 0.4 | 1.1 |
| Urobilinogen (UBG) | 0.5 | 0 | 0.5 | 0.5 | 0 | 0.5 |
| Urine -pH | 6 | 6 | 7 | 6 | 6 | 7 |
| Urinary Bacteria Coun | 19.15 | 6.2 | 100.6 | 22.55 | 6.45 | 131.075 |
| Immunoglobulin G (IgG CSF) | 64.1 | 34.6 | 134 | 58.75 | 36.6 | 118.75 |
| Immunoglobulin A (IgA CSF) | 8.96 | 4.635 | 20.925 | 9.35 | 4.89 | 20.2 |
| Immunoglobulin M (IgM CSF) | 2.34 | 0.8665 | 6.945 | 2.11 | 0.92575 | 6.145 |
| Uric Acid (UA) | 199.5 | 126.25 | 272 | 202 | 129.25 | 271.95 |
| Urinary Crystal Count | 0.1 | 0 | 0.5 | 0.2 | 0 | 0.63 |
| Urinary Mucous Thread Count | 0.27 | 0 | 1.7175 | 0.36 | 0 | 2.0075 |
| Chloride (Cl CSF) | 120.2 | 116.1 | 123.7 | 120.54 | 116.625 | 124.2 |
| Microalbumin CSF (ALB CSF) | 424 | 256.5 | 734.25 | 401.5 | 275.35 | 716.75 |
| Prothrombin Time (PT) | 11.4 | 10.8 | 12.1 | 11.4 | 10.9 | 12 |
| Prothrombin Activity (PTA) | 90.9 | 82.2 | 99.6 | 91.4 | 83.4 | 99 |
| D-Dimer | 0.7 | 0.29 | 2.6825 | 0.93 | 0.34 | 17.0455 |
| Non-lysed Red Blood Cells Ratio | 76.9 | 58.225 | 91.475 | 75 | 59.55 | 88.875 |
| Hepatitis B Surface Antigen (HBsAg) | 0.53 | 0.45 | 0.62 | 0.534 | 0.46 | 0.62 |

**Supplementary Table 3: Indicators Selected by Various Methods**

| RFECV-ADA | Boruta | MI | Spearman |
| --- | --- | --- | --- |
| Mean Corpuscular Volume (MCV) | Mean Corpuscular Volume (MCV) | Immunoglobulin A (IgA CSF ) | Immunoglobulin M (IgM CSF) |
| Lymphocyte Ratio | Hematocrit (HCT) | Immunoglobulin M (IgM CSF) | Immunoglobulin A (IgA CSF ) |
| Hemoglobin (HGB) | Hemoglobin (HGB) | Immunoglobulin G (IgG CSF ) | Microalbumin CSF |
| Mean Corpuscular Hemoglobin (MCH) | Mean Corpuscular Hemoglobin (MCH) | Microalbumin CSF | Glucose CSF |
| Platelet-Larger Cell Ratio (P-LCR) | Red Cell Distribution Width Coefficient of Variation (RDW-CV) | Protein CSF | Immunoglobulin G (IgG CSF ) |
| Glucose CSF | Basophil Ratio | Red Cell Distribution Width Coefficient of Variation (RDW-CV) | Chloride(Cl CSF) |
| Protein CSF | Platelet-Larger Cell Ratio (P-LCR) | D-Dimer | Protein CSF |
| Urobilinogen (UBG) | Mean Platelet Volume (MPV) | Lymphocyte Ratio | D-Dimer |
| Immunoglobulin G (IgG CSF ) | Platelet Distribution Width (PDW) | Mean Corpuscular Hemoglobin (MCH) | Hematocrit (HCT) |
| Immunoglobulin A (IgA CSF ) | Glucose CSF | Mean Platelet Volume (MPV) | Uric Acid (UA) |
| Immunoglobulin M (IgM CSF) | Protein CSF | Platelet-Larger Cell Ratio (P-LCR) | Mean Corpuscular Volume (MCV) |
| Chloride(Cl CSF) | Urobilinogen (UBG) | Basophil Ratio | Urinary Mucous Thread Count |
| Microalbumin CSF | Immunoglobulin G (IgG CSF ) | Platelet Distribution Width (PDW) | Lymphocyte Ratio |
| Prothrombin Activity (PTA) | Immunoglobulin A (IgA CSF ) | Hemoglobin (HGB) | Neutrophil Ratio |
| D-Dimer | Immunoglobulin M (IgM CSF) | Uric Acid (UA) | Lymphocyte Count |
| Non-lysed Red Blood Cells Ratio | Uric Acid (UA) | Urobilinogen (UBG) | Urinary Bacteria Count |
|  | Chloride(Cl CSF) | Mean Corpuscular Volume (MCV) | Neutrophil Count |
|  | Microalbumin CSF | Hematocrit (HCT) | Hemoglobin (HGB) |
|  | D-Dimer | Chloride(Cl CSF) | Non-lysed Red Blood Cells Ratio |
|  |  | Glucose CSF | Platelet Distribution Width (PDW) |

**Supplementary Table 4: Standard Deviations of Model Performance Metrics in 10-Fold Cross-Validation**

| Model | Sets | SEN | SPE | ACC | PPV | NPV |
| --- | --- | --- | --- | --- | --- | --- |
| ENN-XgBoost_V10 | Training set | 0.01 | 0.03 | 0.02 | 0.03 | 0.02 |
|  | Validation set | 0.03 | 0.02 | 0.04 | 0.04 | 0.03 |
| ENN-LR_V10 | Training set | 0.02 | 0.02 | 0.03 | 0.01 | 0.01 |
|  | Validation set | 0.03 | 0.01 | 0.02 | 0.02 | 0.04 |
| SMOTE-XgBoost_V10 | Training set | 0.03 | 0.03 | 0.02 | 0.04 | 0.03 |
|  | Validation set | 0.04 | 0.03 | 0.03 | 0.05 | 0.03 |
| SMOTE-LR_V10 | Training set | 0.01 | 0.04 | 0.03 | 0.02 | 0.02 |
|  | Validation set | 0.03 | 0.03 | 0.02 | 0.03 | 0.04 |
| ENN-XgBoost_V7 | Training set | 0.02 | 0.02 | 0.01 | 0.04 | 0.02 |
|  | Validation set | 0.03 | 0.02 | 0.02 | 0.03 | 0.02 |
| ENN-LR_V7 | Training set | 0.01 | 0.02 | 0.02 | 0.02 | 0.02 |
|  | Validation set | 0.03 | 0.02 | 0.03 | 0.03 | 0.02 |
| SMOTE-XgBoost_V7 | Training set | 0.02 | 0.03 | 0.02 | 0.03 | 0.02 |
|  | Validation set | 0.03 | 0.03 | 0.03 | 0.04 | 0.02 |
| SMOTE-LR_V7 | Training set | 0.02 | 0.02 | 0.03 | 0.03 | 0.01 |
|  | Validation set | 0.03 | 0.02 | 0.03 | 0.02 | 0.02 |

**Supplementary Table 5: Performance of the XgBoost model on data not involved in feature extraction**

| Model | SEN | SPE | ACC | PPV | NPV | AUC |
| --- | --- | --- | --- | --- | --- | --- |
| XgBoost | 77.58% | 76.90% | 77.13% | 77.30% | 78.55% | 0.872(0.835-0.921) |
